# Weil’s Disease in an HIV-Infected Patient

**DOI:** 10.7759/cureus.14241

**Published:** 2021-04-01

**Authors:** Duc B Nguyen, Swethapriya Chaparala, Laurent Morel, Yolin Bueno, Roger D Lovell

**Affiliations:** 1 Graduate Medical Education, Piedmont Athens Regional Medical Center, Athens, USA; 2 Geriatrics, Piedmont Athens Regional Medical Center, Athens, USA; 3 Infectious Diseases, Piedmont Athens Regional Medical Center, Athens, USA

**Keywords:** leptospirosis, hiv infection, weils' disease

## Abstract

Leptospirosis is a zoonosis caused by the spirochete *Leptospira*. Most cases of leptospirosis are mild to moderate and self-limited. The course of disease, however, may be complicated by multiorgan dysfunction with liver and kidney failure causing Weil’s disease. Leptospirosis is also rare among HIV-infected patients. We report a case of an HIV-infected patient with Weil’s disease.

## Introduction

Leptospirosis is caused by *Leptospira* species, which infects humans through exposure to water (flood water, sewage, rivers) and soil contaminated with urine from infected animals [1]. Transmission from the environment to humans occurs through mucous membrane, conjunctiva, and skin abrasions [1,2]. Outbreaks have been reported in certain high-risk activities such as swimming, and boating in floodwater, rivers, or lakes [3-5]. Prolonged immersion or submerging head in water increases the risk even more [2, 3]. Leptospirosis is uncommon in the United States, with approximately 100-150 cases reported annually; the majority of cases were reported in Puerto Rico and Hawaii [2]. The majority of cases in humans present with mild diseases; only 5-10% develop severe manifestations with liver and kidney failure (i.e., Weil’s disease) associated with a high case fatality rate [6].

Leptospirosis is rarely reported among human immunodeficiency virus (HIV)-infected patients in the United States. A case report and review of the literature in 2001 summarized five cases, of which three presented with severe infection [7]. Here we report a severe case of leptospirosis in an HIV-infected patient.

This article was previously presented as a meeting poster at the American College of Physicians (ACP) Georgia Chapter 2020 Virtual Meeting.

## Case presentation

A 51-year-old male with a history of well-controlled HIV infection on antiretroviral therapy presented with a one-week history of fever, chills, and severe myalgia causing functional impairment, left upper quadrant abdominal pain, nausea, vomiting, and diarrhea, dark urine, and decreased urine output. The myalgia was most significant in lower legs bilaterally, and the patient was bed-bound for three days before admission due to pain.

The patient’s past medical history was significant for HIV infection diagnosed in 2005; he was taking Genvoya® (elvitegravir, cobicistat, emtricitabine, tenofovir alafenamide) with adequate viral and immunologic response (CD4 819 cells/mm^3^, undetectable viral load five months prior to admission). He also had a history of resolved hepatitis B infection. His social history was significant for intravenous (IV) drug use, with the most recent use three months prior to admission. He had no recent sick contact or travel outside the current state of residence.

On admission, vital signs showed a heart rate of 109 beats per minute, blood pressure of 130/85 mmHg, and temperature of 37.4 degree Celsius. Clinical examination showed a toxic-appearing male, scleral icterus and jaundice, mild left upper quadrant tenderness without rebound or guarding, bilateral calf tenderness, and multiple abrasions on feet and lower extremities. No conjunctival suffusion was seen. Heart and lung examination was unremarkable, with no sign of meningism.

Laboratory findings on admission showed leukocytosis (white blood cell count of 13,400 cells/mm^3^), elevated aspartate aminotransferase (AST, 154 U/L), alanine aminotransferase (ALT, 67 U/L), total bilirubin (19.2 mg/dL), direct bilirubin (16.88 mg/dL), creatinine (4.2 mg/dL), and creatine kinase (CK, 671 U/L). Hospital course with symptoms, signs, and laboratory findings are presented in Table 1. The patient also developed hypokalemia (2.9 mmol/L) and hypomagnesemia (1.1 mg/dL) on day 3 of hospitalization.

**Table 1:** Hospital course with symptoms and laboratory results

	Max temperature (Celsius degree)	Creatinine, mg/dL (normal: 0.5–1.2 mg/dL)	Bilirubin, mg/dL (normal: 0.2–1.4 mg/dL)	AST, U/L (normal: 12–42 U/L)	ALT, U/L (normal: 7–52 U/L)	Doxycycline
Day 0	39.3	4.2	19.2	154	67	
Day 1	38.2	3.81	18.6	126	59	Yes
Day 2	37.1	3.25	23.6	82	48	Yes
Day 3	36.9	2.38	30.9	54	45	Yes
Day 4	37.0	1.91	28.9	41	39	Yes
Day 5	37.1	1.79	24.5	42	43	Yes
Day 6	36.9	1.57	18.8	44	46	Yes
Day 7	36.9	1.43	15.4	40	48	Yes

In the emergency department, the patient was suspected to have gram-negative sepsis, and piperacillin-tazobactam was started. However, blood culture was obtained, which was negative. Given a clinical picture suggestive of leptospirosis, exposure to water and soil was sought. The patient reported a history of having slid and submerged into a shallow river while outdoors two weeks before onset. Leptospirosis serology was sent and returned positive for IgM (immunoglobulin M) antibody on the fifth day of admission. Microscopic agglutination test and polymerase chain reaction (PCR) for *Leptospira* were not obtained. As part of workup for liver dysfunction on admission, a viral hepatitis panel was checked, which showed negative hepatitis A virus (HAV) IgM antibody, negative hepatitis C virus (HCV) antibody, negative hepatitis B virus (HBV) surface antigen, positive HBV core antibody, and positive HBV surface antibody. Testing for other infections was performed, which showed negative antibody for Lyme disease and negative cytomegalovirus (CMV); however, Epstein-Barr virus (EBV) PCR was positive with a viral load of 8,350 copies/mL (3.92 log). We rechecked the patient’s CD4 count and HIV viral load on admission, and the CD4 was 200 cells/mm^3^ and the viral load was undetectable.

For treatment, the patient was started on doxycycline 100 mg IV twice daily on day 1 of hospitalization, which was later on switched to oral doxycycline 100 mg twice daily on day 3. Piperacillin-tazobactam was stopped. Fluid resuscitation and electrolyte repletion were administered. The patient tolerated antibiotics well without reactions. He showed rapid response to antibiotics with defervescence, improving kidney function and liver function. Bilirubin level continued to increase for three days after doxycycline initiation and then gradually decreased (Table 1). The patient continued to have severe myalgia throughout hospitalization and was discharged after one week of hospital stay. He was followed up via telephonic consultation one week after discharge and reported improvement in myalgia.

## Discussion

We report a case of Weil’s disease in an HIV-infected patient, which has been rarely reported in the United States. Similar to previous case reports, our patient presented with fever, myalgia, jaundice, elevated transaminases, and renal failure; however, unlike our patient, other reported HIV-infected patients with leptospirosis had low CD4 counts ranging from 13 cells/mm^3^ to 60 cells/mm^3^ [7]. Although our patient had normal CD4 counts and undetectable HIV viral load, chronic inflammation and immune reactivation in HIV infection may have contributed to his severe manifestations [8-11]. Leptospirosis presenting with severe sepsis is more common among HIV-infected patients compared to HIV-uninfected patients [12]. Our patient responded favorably to treatment with doxycyline, as the cases in previous reports [7].

There were challenges in the diagnosis of this patient. When the patient was first evaluated, given the fever and multiorgan failure, he was thought to have gram-negative sepsis and broad-spectrum antibiotics were started. His presentation (fever, myalgia, jaundice, liver and kidney dysfunction) led us to inquire about potential exposure to leptospirosis. His history of exposure to river water and skin abrasions made leptospirosis more likely, and serology led to the diagnosis and treatment. Although microscopic agglutination test and PCR for *Leptospira* were not obtained, the clinical manifestations, the exposure, and the positive *Leptospira* IgM should be adequate to confirm leptospirosis. Other differential diagnoses were discussed. Opportunistic infections that may have similar presentations, such as *Mycobacterium avium* complex infection or histoplasmosis, were unlikely due to the patient’s high CD4 count and undetectable HIV viral load. His hepatitis panel excluded acute hepatitis as a cause of liver dysfunction. Lyme disease was unlikely with this clinical presentation and negative serology. Secondary syphilis may also present with fever, myalgia, and elevated liver enzymes; however, the patient did not have characteristic rash, and review of his previous medical records showed a positive rapid plasma reagin (titer 1:1) and positive fluorescent treponemal antibody absorption five months prior to presentation, which made secondary syphilis less likely. CMV and EBV infection may also present with fever, myalgia, and abnormal liver enzymes; however, CMV infection was unlikely due to negative CMV PCR. The patient had positive EBV PCR with a viral load of 8,350 copies/mL. This possibly reflected reactivated EBV infection, and whether it contributed to severe manifestations in our patient remained unclear. One single case of EBV reactivation in leptospirosis was reported in the literature, and the authors postulated that EBV may complicate leptospirosis [13].

During the hospital course, the patient responded well to therapy with rapid resolution of fever and improvement in AST, ALT, and kidney function; however, bilirubin continued to increase after initiation of antibiotics. This phenomenon is commonly seen in leptospirosis and should not be a worrying sign [14,15]. Electrolyte abnormalities seen in our patient (hypokalemia, hypomagnesemia) and prolonged myalgia are also common in leptospirosis [16,17], and physicians should check for those abnormalities and replete if necessary.

## Conclusions

In conclusion, leptospirosis is rare among HIV-infected patients, but it can present with severe manifestations. Leptospirosis should be considered in patients with fever, compatible exposure, and the combination of liver and kidney dysfunction and elevated CK, and appropriate serology testing should be performed. Once leptospirosis is diagnosed, prompt treatment should be started. This case report highlights the importance of detailed history of present illness (including relevant environmental exposures) and thorough physical examination.
